# Evaluation of problem-based learning curriculum implementation in a clerkship rotation of a newly established African medical training institution: lessons from the University of Botswana

**DOI:** 10.11604/pamj.2017.27.13.10623

**Published:** 2017-05-05

**Authors:** Stephane Tshitenge Tshitenge, Chiratidzo Ellen Ndhlovu, Radiance Ogundipe

**Affiliations:** 1 Family Physician, Faculty of Medicine, University of Botswana, Gaborone, Botswana, Department of Family Medicine and Public Health Care, Botswana; 2 University of Zimbabwe College of Health Sciences, Harare, Zimbabwe

**Keywords:** Problem-based learning, curriculum implementation, clerkship, new African medical

## Abstract

**Introduction:**

Problem-based Learning (PBL) curricula, like all curricula, require systematic evaluation as there is a risk of implementing a dysfunctional PBL curriculum. The study intended to evaluate the PBL curriculum delivery from the perspective of the clerkship students at the University of Botswana-Faculty of Medicine.

**Methods:**

A cross-sectional study was conducted among clerkship students in Family Medicine, Paediatrics, Internal Medicine and Surgery. During a 4-week period, each respondent completed weekly a questionnaire based survey tool. The three part questionnaire consisted of demographic data, 'seven-jumps' adapted from a 'typical' PBL tool to evaluate PBL process and 11 items 'adopted 'from the Short-Questionnaire-to-Evaluate-the-Effectiveness-of-Tutors in the PBL tool to evaluate the PBL facilitation with open ended questions at the end.

**Results:**

Of the 81 eligible participants, 89% (n=72) responded. We collected back 141 (49%) forms out of the 288 expected (72 X 4 weeks). PBL first sessions took place all the time only in Family Medicine and in about 75% of the time in Pediatrics but none were conducted in the other disciplines. Overall, they evaluated the PBL process as 'good' (median= 8 /10) and the PBL facilitation as 'very good' (median=9 /10). Students appeared to have differing opinions on the preferred approach to the nature of patient problems that the PBL sessions should be structured around.

**Conclusion:**

Despite students rating PBL process as 'good' and facilitation as 'very good', PBL first sessions were not consistently undertaken.

## Introduction

Problem-based Learning (PBL) is an approach to learning that is used extensively by medical training institutions for the past four decades. A 'typical' PBL consists of a 'seven-jump' procedure that includes in the first session, identifying facts/problems, generating hypotheses, listing needs to know, organizing and prioritizing learning issues/objectives, self-directed learning, and the second session is essentially a feedback session [1]. In a 'hybrid' PBL curricula, prior five lectures per week are reasonable to support PBL learning at the preclinical phase; while in the clerkship, there should be fewer lectures along with seminars, bedside teaching and skills laboratories [2]. The task of the PBL facilitator is to ease the intellectual and relational process for the group [3]. PBL was primarily designed as a learning platform for preclinical phase training and has been extensively studied and adopted in this phase [4]. However, some medical training institutions extend its use to clerkship training [5]. PBL curricula require systematic evaluation as there is a risk of implementing a dysfunctional PBL curriculum [6]. The implementation of PBL is not easy more especially in new institutions; as challenges encountered may include resources, organizational issues and a shift in culture for the academic staff [7]. Literature on the implementation and use of PBL in clinical phase in Sub Saharan Africa is very limited; also literature that assesses the discrepancy in implementation of the PBL curriculum within new institutions is scarce. The University of Botswana-Faculty of Medicine (UB-FOM), a new medical institution that just graduated three cohorts of medical students, adopted a 'hybrid' PBL as a mode of teaching and learning. The UB-FOM's PBL curriculum implementation and facilitation in clerkship was yet to be evaluated, though an early study from UB-FOM conducted on one group of 26 students who were commencing their rotation in emergency medicine attested that more than 90% of students supported the PBL approach [8]. This study intended to evaluate the PBL curriculum delivery from the perspective of the clerkship students at UB-FOM.

## Methods

A cross-sectional study was conducted among year 3, year 4 and year 5 clerkship students rotating in family medicine, paediatrics, surgery and internal medicine departments of the UB-FOM from the 1st October 2015 to the 20th November 2015. Students rotate in small groups of on average 10 students for an 8-week rotation block in these disciplines. The survey instrument, a three-part self-administered and anonymous questionnaire, was constructed as follows; (i) Part I: demographic data, (ii) Part II: CP students' evaluation of the PBL process, and (iii) Part III: CP students' evaluation of the PBL facilitation. Part I of the questionnaire had items that included the clerkship student's class, the rotation block, number of students the PBL group, number of discipline-specific teaching sessions (lectures) in a week, and whether the PBL session 1 and 2 took place. Part II of the questionnaire consisted of seven items adapted from the 'seven-jump' procedure checking whether (1) case scenario provided open-ended problems, (2) unfamiliar terms were identified and clarified, (3) the problem was defined, (4) brainstorming was done, (5) there was a review the definition of the problem, (6) there was formulation of learning objectives, (7) there was contribution to the discussion by all students [1, 5]. Part III of the questionnaire consisted of 11 items adapted from the Short-Questionnaire-to-Evaluate-the-Effectiveness-of-Tutors (SQEET-PBL) tool to evaluate the PBL facilitation [9]. It is one of the instruments to evaluate the facilitation of PBL that has high validity and reliability; it was founded on contemporary constructivist approach theories with five different facilitator competencies which are (i) constructive, (ii) self-directed, (iii) contextual, (iv) collaborative learning and (v) intra-personal behavior [9].

Each of these items was evaluated using a five point Likert scale indicating from (1) strongly disagree to (5) strongly agree with each statement. At the end of the questionnaire, students were asked to provide an overall evaluation of PBL process and facilitation using a scale from 1 (very poor) to 10 (excellent); and an open-ended section that permitted the students to write in short sentence tips to improve the PBL process and the PBL facilitation. We conducted a pilot study using a group of 10 students to test the user friendliness of the questionnaire; and we corrected the tool where it was necessary. In UB-FOM, the first PBL session generally takes place at the beginning a week (Mondays) and the second session is held towards the end of a week (Fridays). PBL sessions are, in a majority of the case, facilitated by academic staff, and in a few cases by residents. During a 4-week period, each respondent completed weekly a questionnaire based survey tool from day one of the PBL week and then submitted it at the end of the week by placing the forms in a specified collection box. All clerkship students rotating in these four disciplines during the study period were asked to participate. We excluded those who did not consent or those who were in a rotation block where PBL sessions were not organised such as MBBS 5 internal medicine. We summarized data in frequency, median ± interquartile range (IQR), tables and figures where it was appropriate. The qualitative data were identified in themes and summarized in frequency. We used a Spearman test to assess the correlation between the quality of PBL process and PBL facilitators. R software version 3.3.1 was used to capture and analyze the data. The level of statistical significance will be taken as below 0.05. For qualitative data, we read through the responses repeatedly to familiarize with the data. Thematic indexes were developed using the Atlas-ti qualitative analysis software. We systematically applied the codes in the thematic index to all the data. Charting was done to bring all data with the same codes together; these were then interpreted by the researchers. We obtained ethical clearance for the study from the University of Botswana (UBR/RES/IRB/1569, X-REF: UBR/RES/ETHI/07). No student or PBL facilitator identifiers, such as names and addresses were captured for purposes of this study. Respondents signed the consent form before embarking on the study, they were also told that they could withdraw at any time they wished. The risk of the study was negligible, since the study did not involve any interventions.

## Results

Out of the 81 eligible CP students in Internal Medicine, Pediatrics, Surgery and Family Medicine, 89% (n=72) responded. Out of the expected 288 forms to return (72 respondents X 4 weeks), 141 (49%) forms were collected back. The majority (n=84; 60%) of the forms were from MBBS year 3 respondents Table 1.

**Table 1:** Clerkship students’ respondents’ distribution per bachelor of medicine class, faculty of medicine, University of Botswana, October to November 2015

Clinical rotation	Class		
	MBBS 3	MBBS 4	MBBS 5
Internal Medicine	28	20	-
Pediatrics	5	-	15
Surgery	35	-	-
Family Medicine	16	-	22
Total	84	20	37
Percentage	60%	14%	26%

**Evaluation of the problem based learning process**: PBL first sessions took place all the time only in Family Medicine and in about three quarters (75%) of time in Pediatrics but none were conducted in the other disciplines. PBL case scenarios derived from patients clerked by students were used in Family Medicine all the time and in Pediatrics in 70% of the cases. In disciplines such as Internal Medicine and Surgery, paper based cases were used as PBL case scenarios. The second session PBL took place in the four disciplines, all the time.Table 2 illustrates scores assigned by students when evaluating the PBL process. Students attributed a median score of 4 (agree) to all the items. Table 3 summarized qualitative data on tips to improve the PBL process. Students expressed that they would like to see PBL first session to take place. 'First session of PBL is very important; I need also to have my learning objectives' (IMYns) Students appeared to have differing opinions on the preferred approach to the nature of patient problems that the PBL sessions should be structured around. Some of the students expressed preference for the case scenarios provided by the facilitators as it was in their pre-clinical phase, while others opted for actual patients clerked by the students. 'The PBL in Internal MED does not analyze the real patients from the wards, but has learning objectives already formulated. This arrangement makes it easy for us to know what to learn and it's a good arrangement' (IMY4) 'Use of case scenarios like in phase 1 stimulate more learning practically than topics' (PY5) 'We should bring into discussion the clerked patient and try to manage the patient' (FMY5) 'Case should be clerked by the student and reviewed by the facilitator before the first session' (PY5).

**Table 2:** Clerkship students evaluating the problem based learning process, University of Botswana, October to November 2015

‘Seven-jump’ PBL procedure	Median (IQR)
The PBL case scenario provides open-ended problems that stimulate inquiry, not a single problem with a well-defined solution.	4 (3-4)
Students identified and clarified unfamiliar terms presented in the scenario; scribe listed those that remained unexplained after discussion.	4 (2-4)
Students defined the problem (s) be discussed; students had different views on the issues, but all were considered; scribe recorded a list of agreed problems	4 (2-4)
Students brainstormed to discuss the problem(s), they suggested possible explanations on basis of prior knowledge, and they drew on each other’s knowledge and identified areas of incomplete knowledge	3 (2-4)
Students reviewed the definition of the problem, the brainstorming and arranged explanations into tentative solutions; scribe organized the explanations and restructured if necessary	3 (2-4)
Students formulated learning objectives and the group reached a consensus on the learning objectives	4 (2-5)
All students contributed to the discussion, regardless of the learning objectives assigned to individuals	4 (4-5)

IQR: interquartile range. Scale: 1= strongly disagree, 2 = disagree, 3 = neutral, 4 = agree, 5 = strongly agree

**Table 3:** Qualitative analysis of clerkship students’ responses on tips to improve problem-based learning (PBL) process and facilitation at the University of Botswana, faculty of medicine, October to November 2015

Suggestions for improvement of the PBL Process	Suggestions for improvement of the PBLfacilitation
First session of PBL to take place	Develop uniform PBL facilitation protocol, train all PBL facilitators to ensure uniformity
Differing opinions on PBL case scenarios: real patients for some students, paper case scenario for others	Encourage collaboration rather than competition among the students, facilitators should encourage the participation of all students
Students to develop their own learning objectives	Facilitators should discourage students reading from notes or books in PBL sessions
	Have a guiding and clarifying style, give the students clear description of your expectations from them right from the first PBL session
	Listen to the students and appreciate that they may have different sources of information, avoid unnecessary intrusions, avoid sensitive remarks
	Develop an objective PBL grading scheme, provide a feedback of grading and PBL session as a formative learning tool, provide effective and constructive feedback after the PBL sessions

**Evaluation of the problem based learning facilitation**: Table 4 shows how students assigned scores per item when evaluating PBL facilitation. The students allotted a median score of 4 (agree) in all the items; one quarter of the students attributed a score of 2 (disagree) in collaborative learning items. For qualitative data on PBL facilitation Table 3, the most recurring themes in the students' responses were the lack of uniformity in the quality of the PBL facilitation. They appreciated the facilitation differently depending on the tutor and rotations. 'The tutors are always knowledgeable, but sometimes lack the skills of teaching or guiding us learn the necessary.'(PY3-1) 'Train staff in this form of curriculum. It is sometimes poorly taught and sometimes okay. Some rotations teach better than others' (PY3-2) Many of the students were against reading from books and notes during the second PBL session as this practice does not augur well for deep learning. 'Allocate questions randomly to students and don't allow reading out feedback as it does not help students learn' (IMY3-1) A guiding, clarifying style and clear expectations without unnecessary intrusions was preferred 'Was involved in the discussion which was good and asked for clarification here and there. Provided a good environment for learning' (FMY3-1) 'From beginning of PBL session states how he prefers his PBL to be' (FMY3-2) 'Allow students to express themselves with minimal intervening' (FMYns) Another concern expressed by the students was the way the PBL sessions were graded. They felt the grading was subjective and suggested that the grading be discussed with the students as a formative feedback that is effective and constructive. 'The facilitator should discuss the grading with us. This will sort of help us know where to improve and how to study'(PY3-3). 'Tutors should at least have a short session after PBL to give a short lecture about the week's topic and how to approach and handle the patient with such condition '(PYns-).

**Table 4:** Clerkship students evaluating the problem based learning facilitation, University of Botswana, October to November 2015

Facilitator competencies		Median (IQR)
Constructive/ Active learning	Summarized what we had learnt in our own words	4 (3-5)
	Searched for links between issues discussed in the tutorial group	4 (3-5)
	Understood underlying mechanisms/theories	4 (4-5)
Self-directed learning	Generated clear learning issues by ourselves	4 (4-5)
	Searched for various resources by ourselves	4 (4-5)
Contextual learning	Applied knowledge to the discussed problem	4 (4-5)
	Applied knowledge to other situations/problems	4 (4-5)
Collaborative learning	Gave constructive feedback about our group work	4 (2-5)
	Evaluated group co-operation regularly	4 (2-5)
Intra-personal behavior	Had a clear picture about his strengths/weaknesses as a facilitator	4 (4-5)
	Was clearly motivated to fulfil its role as a facilitator	4 (4-5)

IQR: interquartile range. Scale: 1= strongly disagree, 2 = disagree, 3 = neutral, 4 = agree, 5 = strongly agree

**Overall clinical phase students' score of PBL process and PBL facilitators**: The students overall responses showed that the students gave a median 'good' score (8, IQR: 7-9) to the PBL process and a median 'very good score' (9, IQR: 7-9) to the PBL facilitation. There was a statistically significant positive correlation between the favorable grade allocated for PBL process and PBL facilitation (rs =0. 67, p=0. 01); and between the students allocated score to the first item assessing the PBL process (PBL case scenario provides open-ended problems that stimulate inquiry, not a single problem with a well-defined solution) and PBL facilitation (rs =0. 24, p=0. 01).

## Discussion

The present study evaluated the PBL curriculum delivery as it was viewed by clerkship students at the UB-FOM. This study found that PBL process varied from one rotation block to another in clerkship the UB-FOM. The first sessions took place all the time only in one rotation block (Family Medicine); while in other rotation blocks it was partly implemented (about three quarters of time in Pediatrics) or not at all in the other disciplines. Although most academic staff undergoes induction training on PBL, the non-experience of some of the academic staff in clerkship may have played a role on the unequal way of implementing the PBL. With the non-implementation of the PBL first session where five of the 'seven-jump' take place, one would wonder if such training platform has created a dysfunctional curriculum with a PBL façade [1, 5]. This study found that there was no uniformity in the way PBL case scenarios were generated; they were derived from clerked patients by students in Family Medicine, while paper cases were used in other rotation blocks. If one group of students commented that having a case paper was easing their learning, another group of students had an opposite point of view as using a real patient stimulated their learning. Although there may be no significant difference between the two approaches, a study found that the use of a real patient as a PBL case scenario was considered significantly more interesting than the paper case by students and it may significantly improve their understanding of the learning objectives and make them feel confident in upcoming patient encounters [10]. Although it may be easy to find a good real patient PBL case scenario during an 8-week rotation block in primary care settings such as Family Medicine, it may not be as easy to find a suitable patient with the week theme scenario in secondary care during an 8-week rotation block. In our study, students felt that overall, the PBL process was 'good' (median score 8, IQR: 7-9). A study from the same institution conducted among Year 3 students rotating in Emergency Department reported that over 90% of respondents were satisfied with PBL and found PBL and Emergency Medicine as an effective combination [8].

In this study, although students felt that overall the PBL facilitation was 'very good' (median score 9, IQR: 7-9), one quarter of the students´ responses allotted a score of 2 (disagree) in collaborative learning items. PBL in UB-FOM is facilitated by faculty, staff and residents in few cases. Collaborative learning as well as stimulating active learning and self-directed learning is considered to be the most important tutor competencies that trigger a higher group functioning, and better achievement in PBL [11]. Academic staff, as a subject expert, may be more likely than non-academic staff (residents) facilitators to display the collaborative competency [12]. Many of the students were against reading from books and notes during the second PBL session as this practice does not augur well for deep learning. By reading from books during feedback session, students give the impression that they are engaged actively in the debate, but that discussion is superficial and do not create embellishment and activation of prior knowledge. As a result PBL becomes a ritual behavior; when such situation arises, the facilitator is to ask students to reflect on their own process. In the study, students suggested that PBL facilitators should adopt a guiding, clarifying style and provide clear expectations without unnecessary intrusions. The students felt that the grading were subjective; they suggested that the grading be discussed with the students as a formative feedback that is effective and constructive. The tool used by UB-FOM to rate the students´ performances during the PBL session has not been validated therefore inappropriate grading of students may be possible and may have contributed to students´ dissatisfaction about the grade. Method of rating students' performances during the PBL session may include tutor´s assessment, peer assessment, and self-assessment; though in summative assessment, peer- and self-assessment marks may be reliable but they lack validity [13]. In our study, there was a statistically significant positive correlation between the favorable grade allocated for PBL process and PBL facilitation (rs =0. 67, p=0. 01) and between the students allocated score to the first item assessing the PBL process (PBL case scenario provides open-ended problems that stimulate inquiry, not a single problem with a well-defined solution) and PBL facilitation (rs =0. 24, p=0. 01). Similar findings were reported in a Japanese study where a rating of 'excellent' regarding case scenarios was associated with excellent tutor evaluations (OR of 12.43 [95% CI: 10.28-15.03) [14].

**Limitations of the study**: The present study did not attempt to assess the proportion of PBL facilitation by non-academic staff tutors and how their PBL facilitation was rated by student. On the other end, we did assess whether there was a difference in rating of the PBL facilitation between lower class students and high class students. As a result of PBL experience, high class students have tended to give a favorable score to facilitators, they become less reliant on their facilitators to make the group functioning and they are more focused on gaining difficult information [15]. Although the response rate to the questionnaire was 89%, which is good in itself, we collected back only 49% of the total forms. This may be a source of non-response bias and may have affected our results as those non respondents may have not had a good experience with the PBL; as a result, our findings should be considered with caution.

**Recommendations**: We recommend a minimum variation of PBL implementation in clerkship within the same institution (UB-FOM). To avoid a possible dysfunctional PBL curriculum, first PBL sessions should take place in all clinical disciplines; but clinical disciplines may choose the approach of PBL case scenario selection as it is not easy to find a good real case during an 8 week rotation block in all disciplines. The institution should organize induction course training of PBL facilitation for non- academic staff (residents), and it should also organize regular PBL facilitation refresher training for both academic staff and non- academic staff that emphasizes on how to provide an effective feedback to students during a PBL session. We also recommend a regular review of the implementation of PBL curriculum in new institutions to detect dysfunctional curriculum on time, and further study that looks at issues such as tools to evaluate students´ performance during PBL sessions.

## Conclusion

Despite students rating PBL process as 'good' and facilitation as 'very good', PBL first sessions were not consistently undertaken. Students seemed not to be satisfied with collaborative learning skills of facilitators. We also recommend a regular review of the implementation of PBL curriculum in new institutions to detect dysfunctional curriculum on time, and methods of evaluation of students' performance during PBL sessions.

### What is known about this topic

- PBL was primarily designed as a learning platform for preclinical phase training and has been extensively studied and adopted in this phase; some medical training institutions extend its use to clerkship training;
- PBL curricula require systematic evaluation as there is a risk of implementing a dysfunctional PBL curriculum.

### What this study adds

- PBL implementation in newly established African clerkship training institutions may include challenges such as non-uniformity of PBL processes within a training institution and the omission of the first PBL session; and this may result in dysfunctional PBL curricula;
- Collaborative learning was one of the competencies that lack in PBL facilitators as reported by one quarter of students; this shows the importance of regular training of facilitators.

## Competing interests

The authors declare no conflicts of interest.
